# A qualitative study of risk and resilience for positive life outcomes in neurodivergence using the WHO ICF

**DOI:** 10.1038/s41598-025-19154-9

**Published:** 2025-10-09

**Authors:** Melissa H. Black, Julie Segers, Emma Moormann, Stephanie Torell, Cecilia Ingard, Sven Bölte

**Affiliations:** 1 Department of Community and Clinical Health, School of Allied Health, Human Services and Sport, La Trobe University, Melbourne, Australia; 2 Department of Women’s and Children’s Health, Center of Neurodevelopmental Disorders (KIND), Centre for Psychiatry Research, Karolinska Institutet and Region Stockholm, Stockholm, Sweden; 3 Department of Philosophy, University of Antwerp, Antwerp, Belgium; 4 Parenting and Special Education Research Unit, Faculty of Psychology and Educational Sciences, KU Leuven, Leuven, Belgium; 5 R2D2-MH Co-Creation Group, University of Antwerp, Antwerp, Belgium; 6 FUNKA Psykologi, Stockholm, Sweden; 7 Department of Social Work and Criminology, Faculty of Health and Occupational Studies, University of Gävle, Gävle, Sweden; 8 Child and Adolescent Psychiatry, Stockholm Health Care Services, Region Stockholm, Stockholm, Sweden; 9 Curtin Autism Research Group, Curtin School of Allied Health, Curtin University, Perth, Australia

**Keywords:** Neurodiversity, International classification of functioning, Autism, ADHD, Neurodevelopmental, Strengths-based, Psychology, Quality of life

## Abstract

Resilience is a dynamic process involving the interaction of multi-systemic individual and environmental factors that operate to protect against adversity and promote positive personal outcomes. Resilience is a topic of interest among groups who commonly experience adversity. Yet, it has received limited attention in the context of neurodivergence (e.g., autism and Attention Deficit Hyperactivity Disorder). This study is part of a larger project investigating the bio-psycho-social factors contributing to resilience in neurodivergence using the World Health Organization’s International Classification of Functioning (ICF). Interviews and focus groups were conducted with 69 neurodivergent individuals and/or their loved ones to explore the factors they believe influence risk and resilience for positive life outcomes and well-being. A deductive qualitative content analysis was employed to extract meaningful concepts from the interviews, which we then quantified by linking concepts to the ICF. A range of bio-psycho-social factors contributing to risk and resilience were identified, particularly in the ICF’s activity, participation, and environmental domains. Key factors included the immediate family, friends, and community members, as well as recreation and leisure, higher-level cognitive functions, and empowerment. Findings, while preliminary, highlight the need to look beyond individual factors alone, emphasizing the variable and context-dependent nature of resilience in neurodivergence.

**Supplementary Information:**

The online version contains supplementary material available at 10.1038/s41598-025-19154-9.

## Introduction

Human resilience is conceptualized in many ways, including as a personality trait, an outcome, and a process^1,2^. Although conceptualizations vary, at a fundamental level, it is concerned with positive psychological and physical health in the face of adversity. Resilience can range from enduring or “doing better than expected” given the circumstances to thriving or flourishing despite adversity^2^. As a process, resilience is not a fixed individual trait but rather a dynamic and ongoing process involving the interaction of multiple internal and external factors that protect against adversity and promote positive functional, well-being, and mental health outcomes^2,3^.

As a process, resilience presents intriguing avenues for support pathways that can promote positive outcomes by targeting modifiable internal and external resilience factors. For this reason, resilience has become a topic of interest in fields such as mental health, where investigation has shifted from solely exploring factors that contribute to ‘psychopathology’ to also identifying those factors that can help maintain mental well-being and aid recovery from challenges^1^. Resilience is also emerging as a topic of interest among other populations who commonly experience adversity, such as racial and ethnic minorities^4^, and LGBTQIA + individuals^5^.

Individuals with divergent neurological functioning resulting from early differences in the structural and functional maturation of the central nervous system, such as those with neurodevelopmental conditions (e.g., autism, ADHD, learning disabilities, intellectual disabilities, and others; henceforth referred to as “neurodivergent”), are another population that experiences adversity, representing a group where exploration of resilience could be transformative. Yet, resilience has received limited attention in this population to date. Neurodivergent individuals experience a range of adversities due to both the nature of neurodivergence and the circumstances in which they are situated. Individuals in these populations can face adversities similar to those of other minority groups, such as discrimination, marginalization, stigma^6,7^, and more adverse childhood experiences^8^. In addition, disability associated with a poor person-environment fit (i.e., a mismatch between individual needs and environmental supports and demands) and genetic and neurobiological factors contributing to an increased likelihood of physical and psychiatric health problems^9–11^, can also contribute to adversity. Emerging evidence further suggests that there may be other experiences not considered adverse by neurotypical standards that also contribute to adversity. For instance, daily hassles may cause more substantial stress^12^, and a broader range of common life events may be experienced as traumatic^13^.

Group-level statistics show that neurodivergent individuals demonstrate poorer outcomes across domains such as mental health, quality of life, education, employment, and mortality^14–17^. Yet, many neurodivergent individuals seem to demonstrate better than expected outcomes despite facing similar adversities. Indeed, there is such considerable heterogeneity in outcomes that accurate predictions of adult outcomes cannot be made based solely on the presence of neurodivergence^18–20^. Resilience thus becomes highly relevant within this population, potentially providing some explanation for the observed heterogeneity and why some neurodivergent people achieve better outcomes than others. Such investigation holds the potential to shift focus from risk and deficit to a more balanced approach that also considers strengths and resources, possibly providing avenues for support pathways that can promote positive outcomes^21^.

Investigating resilience in neurodivergence, while potentially transformative in shaping our understanding and support of neurodivergent people, is complicated by its complex, dynamic, and multi-systemic nature. This complexity necessitates approaches capable of capturing the myriad of factors influencing risk and resilience. The World Health Organization (WHO) International Classification of Functioning, Disability and Health (ICF) provides a framework and classification system appropriate for this purpose. The ICF is a bio-psycho-social framework and comprehensive classification system designed by the WHO to capture the concept of functioning, conceptualized as a dynamic interaction between an individual, their activities and participation, and their environment. The ICF is endorsed by 191 WHO member states, providing a shared language for describing functioning and playing a central role in several international disability policies, assessments, and evaluation processes^22^. The development of Core Sets, or short lists of the ICF, containing only codes relevant to specific conditions or contexts, is also endorsed by the WHO, enabling more direct practical application of the ICF in practice^23^. The ICF thus offers a comprehensive and widely accepted system for investigating the complexities of risk and resilience in neurodivergence, with the generation of Core Sets for resilience in neurodivergence facilitating the development of assessment and support pathways targeting these factors.

The current study is situated within a systematic body of work that seeks to examine and identify those bio-psycho-social factors most important for risk and resilience in neurodivergence and derive ICF Core Sets that include all these pivotal risk and resilience factors^24^. Our previous work using the ICF to identify pertinent risk and resilience factors in neurodivergence included a scoping review of prior research^25^, and a survey of professional perspectives on risk and resilience factors in neurodivergence^26^. Although this work provides some initial insights into the nature of resilience in neurodivergence, we currently know very little about what neurodivergent individuals and those close to them perceive as important for risk and resilience. Only neurodivergent individuals and those close to them have access to lived experience^27^, providing unique additional insights into the nature of risk and resilience from primary and secondary perspectives. For this reason, we sought to explore the perspectives of neurodivergent individuals and their loved ones to identify individual capacities, environmental factors, and factors related to activities and participation they perceive as important for resilience in neurodivergence.

## Results

**Fig. 1:**
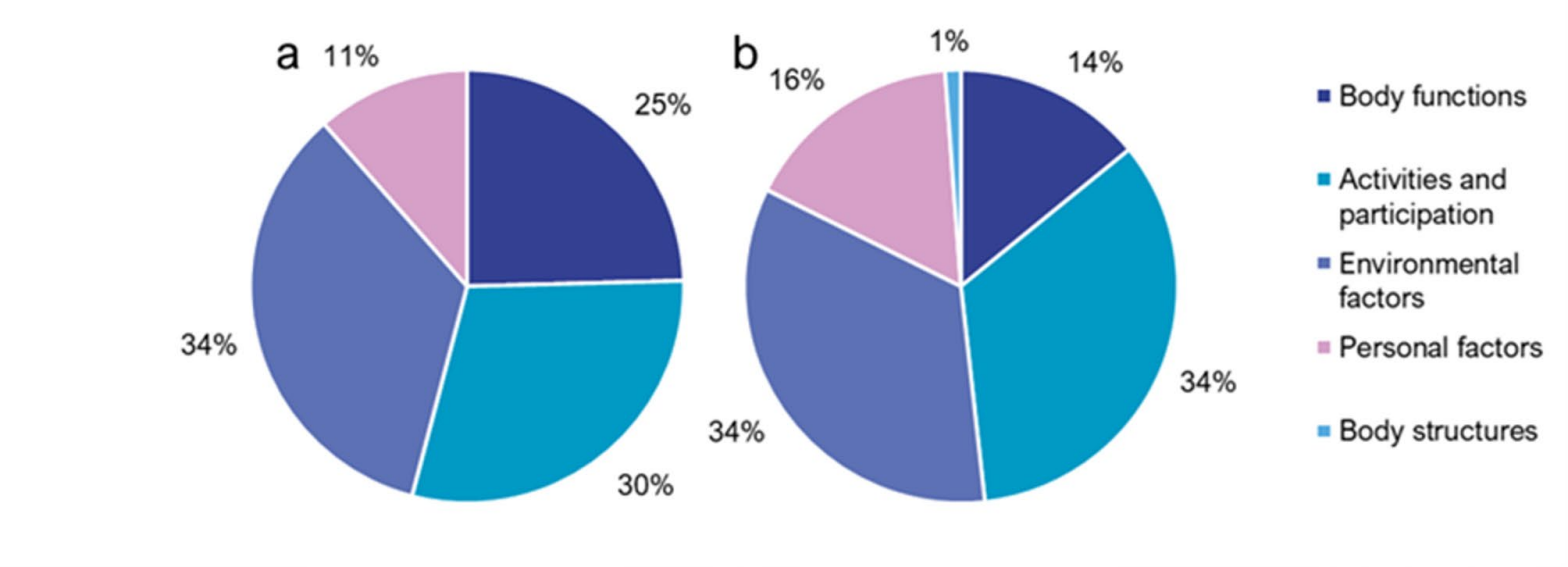
Distribution of ICF codes for Risk (**a**) and Resilience (**b**).

A total of 4202 meaningful concepts were extracted from the 69 interviews. Each interview generated a median of 60 meaningful concepts (range: 9–192 concepts). These meaningful concepts were linked to a total of 4999 codes, including 3942 ICF codes and 586 personal factor codes. There were several instances in which HC codes (k = 26), NC codes (k = 358), and ND codes (k = 78) were also applied. Of the 4528 ICF and personal factor codes applied, 198 were unique, with 106 of these codes appearing in at least 5% of the interviews (range: 6–74%). ). As shown in Fig. 1 codes from the environmental, and activities and participation domains were most frequently identified as contributing to risk and resilience. More body functions were identified as risk factors (25%) than resilience factors (14%).

### Body functions

Body functions were identified as both risk and resilience factors. Fifteen body function codes were identified as contributing to risk, and 12 were identified as contributing to resilience (Table 1). Most codes within the body functions domain were identified as representing both risk and resilience (k = 10), indicating that variations in functioning across these domains determine whether the specific function contributes to risk or resilience. For risk, three of the eight body function chapters were represented, including mental functions (b1), sensory functions and pain (b2), and functions of the digestive, metabolic, and endocrine systems (b5). For resilience, two body chapters were represented: mental functions (b1) and neuromusculoskeletal and movement-related functions (b7). Across both risk and resilience, the mental functions chapter (b1) was the most frequently linked.

**Table 1:** Absolute (f) and relative (%) frequencies of ICF (-CY) body function codes linked to risk and resilience.

ICF code	*f*	%
Risk		
b130 Energy and drive functions	21	30
b152 Emotional functions	19	28
b156 Perceptual functions	19	28
b164 Higher-level cognitive functions	15	22
b126 Temperament and personality functions	12	17
b160 Thought functions	12	17
b144 Memory functions	9	13
b125 Dispositions and intra-personal functions	8	12
b230 Hearing functions	8	12
b122 Global psychosocial functions	5	7
b134 Sleep functions	5	7
b140 Attention functions	5	7
b515 Digestive functions	5	7
b265 Touch function	4	6
b299 Sensory functions and pain, unspecified	4	6
Resilience		
b164 Higher-level cognitive functions	37	54
b125 Dispositions and intra-personal functions	30	43
b126 Temperament and personality functions	30	43
b130 Energy and drive functions	17	25
b156 Perceptual functions	12	17
b134 Sleep functions	10	14
b117 Intellectual functions	9	13
b122 Global psychosocial functions	8	12
b144 Memory functions	7	10
b152 Emotional functions	6	9
b730 Muscle power functions	5	7
b160 Thought functions	4	6

Organized in descending order within risk and resilience categories.

Higher-level cognitive functions (b164) were frequently linked to both resilience (54%) and risk (22%). Here, participants identified problem-solving (b1646), insight (b1644), and cognitive flexibility (b1643) as important for their resilience. For example, one participant shared in relation to insight: “I guess it all comes back to self-awareness and knowing, knowing your limits and knowing who you are and what is going to fill your cup, what’s going to drain you completely and what, what your capacity is to handle different situations” (Participant [P]36; neurodivergent person and caregiver of neurodivergent person). On the other hand, when participants believed they had difficulties in these areas, they were perceived to be risk factors. Likewise, energy and drive functions (b130) were also frequently identified as both risk (30%) and resilience (25%) factors. Participants reported that low energy (b1300) and motivation (b1301) were barriers to engaging in major life areas and coping with difficulties. Conversely, high energy and motivation could facilitate engagement and were necessary for perseverance.

Temperament and personality functions (b126) and dispositions and intrapersonal functions (b125) were also commonly identified as contributing to both resilience and risk, but were more frequently associated with resilience. Commonly identified resilience factors within these areas included optimism (b1265) and persistence (b1254). One caregiver shared:

And his own inner temperament, like I said, he’s always just been persistent about what the job is at hand, going to school, whatever that may be. He’s persistent about going to work. Like he’s not one of those kids who makes excuses why he can’t go. Persistence, I guess, is part of it too. And that’s something innate in him (P11; caregiver of a neurodivergent person).

Responses associated with temperament and personality (b126) and dispositions and intrapersonal (b125) functions seemed to indicate that their function as either a risk or resilience factor fluctuated over time. For instance, some adult participants suggested that agreeableness (b1265) contributed to resilience in their childhood but could contribute to risk in adulthood by interfering with self-advocacy.

Functions related to the senses (b230, b265, b299) and the perception of sensory information (b156) were commonly identified as risk factors, primarily due to their contribution to feelings of overwhelm and their impact on participation in major life areas. One participant shared: “I hear everything, bells, fluorescent lights, it sounds almost silly but sometimes I can feel that I hear the electricity, as it were. Everything goes to it. I don’t see any advantage in that, it tires you out” (P56, neurodivergent person and caregiver of neurodivergent person). However, they also went on to say: “you can learn, I have been able to control it and choose to put on music instead”. Some other participants identified certain perceptual strengths, such as visual perception (b1561), that acted as resilience factors through their contribution to positive outcomes.

Emotion functions (b152), most often referring to emotion regulation abilities (b1521), were also frequently identified as contributing to risk (28%). A smaller percentage (9%), however, identified that emotion functions could also contribute to resilience; for instance, one participant shared: “I've been thinking about one thing a lot, that I think being able to feel what you feel. Where, just to learn to localize what the body feelings are and stuff like that. I think that’s a very important thing… To get some control over it, even if you get strong feelings” (P58, neurodivergent person).

### Activity and participation

Codes in the activity and participation domain were associated with both risk and resilience. For risk, seven of the nine activity and participation chapters were represented, including learning and applying knowledge (d1), general tasks and demands (d2), self-care (d5), domestic life (d6), interpersonal interactions and relationships (d7), major life areas (d8) and community, social and civic life (d9). For resilience, eight of the nine chapters were represented; learning and applying knowledge (d1), general tasks and demands (d2), mobility (d4), self-care (d5), domestic life (d6), interpersonal interactions and relationships (d7), major life areas (d8) and community, social and civic life (d9). In total, 18 codes in this domain were associated with risk, and 28 codes were associated with resilience. Thirteen codes were identified as representing both risk and resilience (Table 2).

**Table 2:** Absolute (f) and relative (%) frequencies of ICF (-CY) activity and participation codes linked to risk and resilience.

ICF code	*f*	%
Risk		
d230 Carrying out daily routine	17	25
d240 Handling stress and other psychological demands	17	25
d750 Informal social relationships	12	17
d820 School education	12	17
d740 Formal relationships	11	16
d920 Recreation and leisure	10	14
d161 Directing attention	9	13
d760 Family relationships	9	13
d720 Complex interpersonal interactions	8	12
d210 Undertaking a single task	6	9
d845 Acquiring, keeping and terminating a job	6	9
d570 Looking after one’s health	5	7
d640 Doing housework	5	7
d710 Basic interpersonal interactions	5	7
d770 Intimate relationships	5	7
d177 Making decisions	4	6
d220 Undertaking multiple tasks	4	6
d850 Remunerative employment	4	6
Resilience		
d920 Recreation and leisure	49	71
d760 Family relationships	39	57
d750 Informal social relationships	33	48
d570 Looking after one’s health	32	46
d230 Carrying out daily routine	23	33
d770 Intimate relationships	19	28
d910 Community life	17	25
d163 Thinking	13	19
d240 Handling stress and other psychological demands	13	19
d450 Walking	13	19
d161 Directing attention	12	17
d850 Remunerative employment	12	17
d455 Moving around	11	16
d740 Formal relationships	10	14
d132 Acquiring information	9	13
d630 Preparing meals	8	12
d820 School education	8	12
d720 Complex interpersonal interactions	7	10
d855 Non-remunerative employment	7	10
d930 Religion and spirituality	7	10
d115 Listening	6	9
d175 Solving problems	5	7
d210 Undertaking a single task	5	7
d650 Caring for household objects	5	7
d810 Informal education	5	7
d155 Acquiring skills	4	6
d660 Assisting others	4	6
d799 Interpersonal interactions and relationships, unspecified	4	6

Organized in descending order within risk and resilience categories.

Recreation and leisure (d920) was the most frequently occurring code in this domain, linked to 71% of the interviews. This captured primarily hobbies (d9204), crafts (d9203), sports (d9201), and socializing (d9205), which were important for promoting resilience. A smaller percentage (14%) of interviews also identified these aspects as contributing to risk, primarily due to reduced ability to engage in these activities.

Engaging in other productive occupations, such as school education (d820), informal education (d810), remunerative employment (d850), and non-remunerative employment (d855), also contributed to resilience. In relation to employment, one participant stated: “yeah right now I think my work is very interesting and helpful itself” (P10, neurodivergent person). While more frequently identified as contributing to resilience, these occupations were sometimes associated with risk, particularly school education (d820) and employment (d845, d850), where increased demands and difficulties participating in these domains represented risks for negative outcomes. Acquiring, keeping, and terminating a job (d845) was uniquely identified as a risk factor, primarily due to stressors related to the job interview process and difficulty gaining employment.

Handling stress and psychological demands (d240) was identified as a risk factor by a quarter of participants (25%), while 19% associated this code with resilience. Generally, participants reported challenges in managing the psychological demands required to carry out tasks involving stress, pressure, or responsibility, which contributed to risk and eroded their resilience. On the other hand, some participants reflected that they operated well under high-stress situations. One participant reflected, “I don’t panic in crisis situations. So that is a form of resilience. Whether I suffer from it afterwards is another matter, but at that moment I can keep a reasonable overview, so that is a positive thing” (P48, neurodivergent person).

While some identified carrying out the daily routine (d230) as contributing to resilience, it was more frequently associated with risk. Other related codes, including doing housework (d640), undertaking single tasks (d220), and making decisions (d177), were also only associated with risk. Common to these items were reported difficulties in these areas, which were reported to contribute to stress through increased mental and physical demands. Conversely, caring for household objects (d650) was identified as a resilience factor by a small portion of interviews, however, this referred exclusively to feeding and caring for pets.

Relationships, particularly family relationships (d760), informal social relationships (d750), intimate relationships (dd70), and less frequently formal relationships (d740), were important resilience factors, however, they were sometimes also identified as risk factors, primarily when individuals perceived that they had difficulties forming and maintaining relationships with the various groups. Relatedly, both basic (d710) and complex (d720) interpersonal interactions, which capture various social skills, were also identified as risk factors. However, a small percentage of participants identified complex interpersonal interactions as contributing to resilience, mostly when they felt they had strengths in this area.

Looking after one’s health (d570) was a resilience factor linked to nearly half of the interviews, usually referring to managing diet and fitness (d7501). Participants shared the importance of eating healthily and exercising, with many reporting that feeling healthy led to feeling happier and more able to cope with challenges. One participant shared: “I think exercise for me has always been a way to come back to center and get rid of the cobwebs and all those things you’re thinking about that you don’t need to think about or you’re overthinking and just to get rid of all that stuff out of life and come back and just go, yeah, this is what life’s about” (P31, neurodivergent person). Managing comfort and energy resources was also related to looking after one’s health. Emphasized by one participant:

I should also like be really careful about my resources we’re talking about spoon theory, if suddenly I spend all my spoons during the day and have no more spoons and that in the evening something bad happens I cannot, I don’t have the resources to take care of that so my resilience fades (P6, neurodivergent person).

### Environmental factors

Environmental factors represented both risk and resilience, and all chapters were represented: products and technology (e1), natural environment and human-made changes to the environment (e2), support and relationships (e3), attitudes (e4), and services, systems, and policies (e5). Twenty-nine codes for resilience and 21 for risk were identified (Table 3). Most codes contributed to both risk and resilience (k = 18).

**Table 3:** Absolute (f) and relative (%) frequencies of ICF (-CY) environmental codes linked to risk and resilience.

ICF code	*f*	%
Risk		
460 Societal attitudes	23	33
e585 Education and training services, systems and policies	23	33
e455 Individual attitudes of other professionals	20	29
e499 Attitudes, unspecified	20	29
e425 Individual attitudes of acquaintances, peers colleagues, neighbours and community members	17	25
e465 Social norms, practices and ideologies	16	23
e310 Immediate family	14	20
e325 Acquaintances, peers colleagues, neighbours and community members	14	20
e580 Health services, systems and policies	13	19
e399 Support and relationships, unspecified	12	17
e410 Individual attitudes of immediate family members	12	17
e250 Sound	11	16
e330 People in positions of authority	11	16
e360 Other professionals	11	16
e430 Individual attitudes of people in positions of authority	11	16
e110 Products or substances for personal consumption	8	12
e240 Light	7	10
e355 Health professionals	6	9
e450 Individual attitudes of health professionals	6	9
e165 Assets	5	7
e320 Friends	5	7
Resilience		
e310 Immediate family	51	74
e320 Friends	43	62
e325 Acquaintances, peers colleagues, neighbours and community members	35	51
e360 Other professionals	32	46
e115 Products and technology for personal use in daily living	29	42
e110 Products or substances for personal consumption	22	32
e585 Education and training services, systems and policies	21	30
e399 Support and relationships, unspecified	19	28
e298 Natural environment and human-made changes to environment, other specified	18	26
e330 People in positions of authority	18	26
e355 Health professionals	18	26
e410 Individual attitudes of immediate family members	18	26
e580 Health services, systems and policies	17	25
e350 Domesticated animals	13	19
e425 Individual attitudes of acquaintances, peers colleagues, neighbours and community members	11	16
e315 Extended family	10	14
e455 Individual attitudes of other professionals	10	14
e165 Assets	8	12
e430 Individual attitudes of people in positions of authority	8	12
e240 Light	7	10
e420 Individual attitudes of friends	7	10
e570 Social security services, systems and policies	7	10
e499 Attitudes, unspecified	6	9
e555 Associations and organizational services, systems and policies	6	9
e130 Products and technology for education	5	7
e460 Societal attitudes	5	7
e575 General social support services, systems and policies	5	7
e260 Air quality	4	6
e590 Labour and employment services, systems and policies	4	6

Organized in descending order within risk and resilience categories.

Support from a range of different individuals was frequently identified as important for resilience. Immediate family (e310), friends (e320), and acquaintances, peers, colleagues, neighbors, and community members (e325) were the top three most frequently identified resilience factors. Family, in particular, was the most frequently identified resilience factor, linked to nearly three-quarters of the interviews. Other professionals, most commonly teachers (e360), people in positions of authority (e330), health professionals (e355), and to a lesser extent the extended family (e315), were also identified as important for resilience, as were pets (e350). In referring to the role of these supports, one participant shared, “There are some people who actually, in my life, who can actually make me feel more empowered, make me feel stronger and make me do things that maybe I didn’t want to do or not doing” (P3, neurodivergent person). However, these groups were sometimes also identified as risk factors, primarily when they failed to provide adequate emotional or practical support. This highlights the dual potential for groups to act as both risk and resilience factors:

It starts from school because you can imagine a teacher calling you dumb and stupid, it will be really hard to recover from that, you will always feel dumb and stupid. But when a teacher empowers you right from the start and tells you you’re smart, you can do this and this, you know they feel good (P30, caregiver of a neurodivergent person).

The attitudes of various groups were also found to contribute to both risk and resilience, however, they seemed to be more often associated with risk. For example, the attitudes of other professionals such as teachers (e455), acquaintances, peers, colleagues, neighbors, and community members (e425), the immediate family (e410), people in positions of authority such as employers (e430), and health professionals (e450), were all identified as contributing to risk. In most cases, participants perceived that these groups had negative attitudes and poor understanding of neurodiversity and support needs. In contrast, some participants perceived that the attitudes of these groups could also contribute to resilience when they had a positive attitude and understanding of neurodiversity.

More broadly, societal attitudes (e460) were identified as a risk factor in 33% of the interviews, while social norms, practices, and ideologies were identified as risk factors in 23% of the interviews. Participants described how broad societal attitudes and understanding toward neurodivergence and expectations to conform to neurotypical standards negatively impacted their quality of life and functioning, in turn eroding resilience and increasing risk. One participant shared: “It just doesn’t seem like a world where you get through it unscathed with ADHD. You have to sit and focus—just good luck with that. Like if you’re not doing that it’s probably a ‘you’ problem, that’s kind of what I was told” (P13, neurodivergent person). Another shared: “Even society…you know these myths that it’s caused by vaccines, like you picked it up like a cold, you know, you did something wrong, you did something wrong in your life and now your brain is broken, or your brain is broken because you have autism” (P1, neurodivergent person). A caregiver in the African Region further stated that understanding of dyslexia was poor in their country, affecting overall societal attitudes and access to resources, potentially pointing to cultural differences in how risk and resilience factors may operate. Conversely, a small percentage of interviewees (7%) reflected that the society they lived in was potentially more accepting and accommodating towards neurodiversity than other societies, identifying this as a resilience factor. Some also reflected that they felt “lucky” that their way of functioning was valued by society: “I know people who are, you know, intelligent in other ways that society doesn’t value. So, I think I’ve been lucky that way” (P19, neurodivergnet person).

A range of services, systems, and policies, primarily those related to education (e585) and health (e580), were identified as contributing to both risk and resilience. A small proportion identified labor and employment services, systems, and policies (e590) as a resilience factor. Generally, participants found that services and systems were ill-equipped to meet their needs but also expressed that certain factors, such as the availability of individual education plans, could support positive outcomes. Other factors in the physical environment also influenced risk and resilience. Participants reported that access to nature (e298) and sunlight (e240) were important for resilience through their impacts on quality of life and coping. One participant shared: “The light. For example, for me, personally, the light in [place] is a lot, it’s a lot brighter, it’s a lot, gives you a lot happier mood. In environments, things like light, sun, darkness, also impacts the quality of life” (P3, neurodivergent person).

### Body structures

The Structure of the brain (s110) was the only body structure code that appeared in 5% or more of the interviews. It was identified as contributing to resilience in 19% of the interviews. This code likely appeared because it was specifically prompted by the question, “What parts of your body affect your ability to live a good life? (Body structure).” Many participants responded, “my brain” while others described how the structure of the brain influenced its function and, consequently, their resilience:” Yeah. But like my brain, that’s, I guess, my neurologic system, my brain is also providing all the things that we’ve talked about in terms of some of those resilience skills” (P18, neurodivergent person).

### Personal factors

Seven personal factor codes related to risk and 14 for resilience were identified from the interviews. Six codes were identified as both risk and resilience factors, with only one unique code identified for risk (i540—ethnic affiliation). Across both risk and resilience, two of the six chapters were represented: i4) attitudes, basic skills, and habits, and i5) life situation and socioeconomic/cultural factors. Identified risk and resilience factors in this domain are displayed in Table 4.

**Table 4:** Absolute (f) and relative (%) frequencies of ICF (-CY) personal factor codes linked to risk and resilience. Organized in descending order within risk and resilience categories.

ICF code	*f*	%
Risk		
i411 Attitude towards one’s own self	14	20
i515 Residential status	9	13
i422 Attitude toward work	7	10
i436 Empowerment	7	10
i418 Attitude toward assistance by others	5	7
i525 financial status	4	6
i540 Ethnic affiliation	4	6
Resilience		
i436 Empowerment	35	51
i416 Attitude toward health, disease and disability	31	45
i515 Residential status	21	30
i411 Attitude towards one’s own self	18	26
i422 Attitude toward work	14	20
i418 Attitude toward assistance by others	12	17
i428 Attitudes, other specified	11	16
i421 Attitude toward education	10	14
i410 World view	9	13
i425Attitude toward social environment/society	9	13
i433 Methodical skills	9	13
i413 Satisfaction with life	7	10
i525 Financial status	7	10
i520 Employment status	4	6

The most frequently identified resilience factor was empowerment (i436), which refers broadly to the ability to perceive one’s strengths and weaknesses, stand up for personal goals, advocate for goals, needs, and boundaries, and develop strategies to achieve goals. Empowerment was also identified as a risk factor, but only when individuals perceived themselves as having low empowerment. Some participants reported that empowerment developed over time, shifting from low self-advocacy to strong self-advocacy, often driven by increased self-awareness after receiving a diagnosis. One participant shared their journey developing empowerment:

But I guess that comes from the people-pleasing part of the ADHD and things like that. Oh, I can do that - so, I should say yes, I will do that. And actually, learning to say no. I think it’s probably part of making me a bit more resilient is actually having the confidence to say "no, that’s not in my best interest right now” to get me through this situation. (P33, neurodivergent person).

Other personal factors included attitudes towards one’s own self (i411) and attitudes toward health, disease, and disability (i416), which were often discussed in tandem. Participants described how their perception of their diagnosis influenced their self-perception, particularly related to self-efficacy:

I feel much more resilient after my diagnosis than before, because I now know that the things that I did before, that wasn’t so obvious at all. And that I really should be proud of myself. So, resilience for me also does correlate with pride and with self-esteem actually as well. Self-esteem, yes, giving yourself a pat on the back, those things, that’s part of it for me as well (P43, neurodivergent person).

Ethnic affiliation (i540) was the only risk factor not also identified as a resilience factor. Here, a small proportion of participants perceived that their ethnic affiliation was a risk factor due to stigma and attitudes of others, which could contribute to negative well-being and quality of life outcomes. For instance, one participant shared how experiences of racism and micro-aggressions wore down their resilience over time:

Next thing you hear, ‘go back to where you came from, my country’s not accepting of your type.’ You hear those things on a day-to-day, plus those little micro-aggressions, you don’t know how much those things get under your skin and before you know it, you’re flinging a stapler at someone (P17, neurodivergent person).

### Uncoded concepts

Several other concepts influenced risk and resilience but were not covered by the ICF(-CY) or personal factor classification system. Here, we summarize the most salient concepts derived from phase one of the data analysis process (qualitative content analysis), based on either the frequency with which participants mentioned them or their impact.

Several participants referred to co-occurring physical and mental health conditions that could erode their ability to cope and their well-being over time, thus contributing to risk. One participant shared how their mental health condition impacted their ability to cope with stress: “You know, with my Bipolar, like I have my, my ability to handle stress has just, is not really, is not really the best” (P9, neurodivergent person). Another participant discussed how their chronic conditions could contribute to risk:

Yeah, like having four different chronic diseases is not fun, two of which can kill me. I have Crohn’s and interstitial cystitis which is painful bladder syndrome um and endometriosis and congestive heart failure, and congestive heart failure and the Crohn’s is definitely going to try to kill me at some point (P1, neurodivergent person).

This participant went on to further discuss how medications for these conditions could also take a toll on their resilience: “you know and the medications I take uh eight different medications just for the congestive heart failure and the uh um you know sometimes you don’t sleep well because of the pain uh you know the drugs have weird side effects uh and you know that affects you” (P1, neurodivergent person).

A commonly identified risk factor for participants, particularly autistic participants, but also some with ADHD, was masking. One caregiver participant shared how their daughters’ masking at school was a substantial risk factor for mental well-being and functioning:

Masking, in my view, is not a good thing...I just could see the pain as she’s walking when I pick her up, I knew soon as I pick her up at school...straight away you know and then something’s gonna be broken or some harm or something will happen you know, so that’s the sort of things of masking (P32, caregiver of neurodivergent person).

Other risk factors included adverse life experiences where participants shared traumatic experiences, including sexual assault, that acted as long-term factors contributing to negative mental well-being and functioning. In relation to resilience, some transgender participants reported that living in alignment with their identified gender was important for their resilience and overall well-being. Although relevant to only a few participants, it was deemed necessary to report due its significance as outlined by one participant:

Just the 1 year into testosterone, like, I actually like the way that my voice sounds now, and I’m starting to have facial hair, which is really nice. So those kinds of things just that yeah, because being a guy is important for living a good life…living in a way that matches my gender is really important (P18, neurodivergent person).

## Discussion

This study aimed to examine factors influencing risk and resilience from the perspectives of neurodivergent people and their families with the intention of informing ICF Core Sets for risk and resilience in developmental diversity. The factors identified by participants included many that align with those of previous studies in other populations, such as mental health^3^, potentially indicating that there are shared resilience factors across different populations. On the other hand, insights from neurodivergent individuals also point to several factors that may be more salient for neurodivergent populations, such as empowerment, attitude towards health and disability, and environmental influences such as the attitudes of others.

Generally, relatively few ICF codes were identified within a majority of interviews. Immediate family (e310), friends (e320), acquaintances, peers, colleagues, neighbors, and community members (e325), empowerment (i436), recreation and leisure (d920), family relationships (d760), and higher-level cognitive functions (b164) are the only codes identified in over 50% of interviews. These factors may have been most frequently identified because they represent more universal experiences, whereas other factors may reflect greater inter- and intra-individual nuance. Rather than as an individual trait, recent discourse surrounding resilience frames it as a multi-dimensional concept influenced by broader social and environmental factors^1,3^. Our findings would seem to support this, with social support and relationships playing a central role, with four of the seven factors identified in more than 50% of the interviews relating directly to support and relationships. Social supports are consistently identified as key protective factors across a range of populations^25,28,29^. Indeed, a recent review of resilience in neurodivergent individuals found that it identified a range of internal and external influences, with social support being one of the most commonly identified resilience factors^25^. Likewise, factors like self-determination, empowerment, choice and making a meaningful contribution have also been identified previously in the extant literature^25^. Our findings thus appear to align with existing literature. However, it is important to note that most previous studies have been guided by researcher-defined frameworks, rather than by factors identified as necessary by neurodivergent individuals themselves. Beyond the more commonly identified factors, several others were identified by only a few participants, possibly reflecting the heterogeneity of our sample. The high inter-individual variability in risk and resilience factors suggests that, while there appear to be some potentially more core or “shared” risk and resilience factors, many more may be individually driven, indicating a need for person-centered approaches when identifying and targeting resilience-enhancing factors.

At the same time, interviews demonstrated the context-dependent nature of risk and resilience. For instance, most identified factors were reported to contribute to both risk and resilience, with their functions described as differing based on the individual’s environment, cultural context, and personal history, including their developmental stage. In this way, particular factors may act as protective or promotive in some instances but as risk factors in others, a finding that broadly aligns with conceptual models of resilience developed by Ungar and Theron^3^. Environmental factors, in particular, were perceived as acting both as risk and resilience factors themselves, but also as shaping the operation of others. For instance, participants suggested that a lack of understanding from social contexts was both a direct and a more indirect risk factor, as it encouraged masking, which in turn contributed to risk. Environments were also reported to interfere with the function of resilience-enhancing factors such as engagement in employment. Policies, services, and systems related to healthcare, education, and employment were perceived as important for facilitating engagement with other resilience-inducing factors, such as education and employment, and for buffering against risk factors like mental and physical health conditions. However, for many participants, these services, systems, and policies failed to meet their needs, rendering them risk factors. Diagnosis also seemed to be a key moment for several risk factors to transition into resilience factors. For many participants who reported receiving a diagnosis later in life, this event served as a pivoting point, primarily through changing their understanding and acceptance of their own self, their strengths, difficulties, and needs. The impact of diagnosis appears to align with previous research, suggesting that a diagnosis can positively influence self-understanding and acceptance^30^.

The lived experience perspectives of factors important for risk and resilience captured in the current study largely echo those of professionals^26^ and previous literature^25^, indicating that there may be some consensus between groups on the factors contributing to risk and resilience in neurodivergence. However, there are some key differences and nuances that lived experience perspectives contribute. Although people with lived experiences emphasized the importance of societal attitudes and attitudes of the community, family members, and other professionals (< 33%), these factors were only identified by < 14% of professionals in our previous study^26^. Likewise, support from family, friends, and other groups, including health professionals, was less frequently identified by professionals as influencing risk and resilience than by participants in the current study. Thus, compared to the results of the current study, there are some indications that professionals may underestimate the importance of support and attitudes from others and, critically, potentially underestimate the impact of their own role and attitudes.

Professionals may also underestimate the role of other risk and resilience-inducing factors, particularly those that may be considered more salient or unique for neurodivergent populations. For instance, empowerment was identified by just over half (51%) of the neurodivergent participants as important for resilience, compared to 16% of professionals. Attitudes toward health, disease, and disability were also frequently identified as influencing resilience by neurodivergent individuals and their loved ones (45%), whereas this was identified in less than 5% of the responses of professionals. Given that neurodivergent individuals and their loved ones identified these factors as important for their resilience, and as they were perceived as influencing other resilience-inducing factors, such as attitude towards oneself, it suggests that greater focus should be placed on providing neurodiversity-affirmative education about one’s diagnosis and providing opportunities for developing self-advocacy and empowerment skills^31^. Overall, comparisons between lived experience experts and professionals in our previous study demonstrate the importance of co-creating with neurodivergent individuals and including their voices when exploring factors that may facilitate or hinder positive outcomes. As codes in the activity and participation and environmental domains were most frequently associated with risk and resilience, the findings point to a need to shift focus from purely biological and individual mechanisms of resilience towards environmental modifications and promoting participation in everyday life domains. For example, in the environment, findings indicate a need for greater societal understanding and acceptance, improved access to resources, and services, and systems and policies that facilitate meaningful engagement in key life areas. Further, as those surrounding neurodivergent people, such as families, healthcare professionals, educators, and employers, seem to play a critical role in influencing an individual’s resilience, greater education and training for these groups on neurodivergence could be beneficial. In the activity and participation domain, supporting individuals to engage in recreational and productive activities, participate in their communities, and build skills to manage stress and navigate daily routines seems important for promoting resilience.

Although activity and participation and environmental domains were frequently associated with risk and resilience, insights from our participants also identified several individually driven mechanisms, such as emotion regulation or sleep, that may also benefit from targeted intervention and support. In addition, a significant number of health condition codes that are not linked to the ICF system but are nevertheless important to capture were highlighted by participants as increasing the risk for poor mental health, well-being, and functioning outcomes. Participants frequently identified mental and somatic health conditions as eroding resilience and contributing to risk. These conditions may be particularly crucial to treat, given their high occurrence in neurodivergent populations and their potential compounding impact on outcomes^32,33^.

We had a few participants identifying as transgender as well as those identifying as belonging to ethnic minorities. Although only a small proportion of the sample, there appeared to be indications that belonging to intersectional groups (e.g., LGBGTQIA+, ethnic minorities) increased or multiplied the experience of adversity. These findings largely reflect broader literature that suggests that multiple intersecting identities can compound negative experiences and psychological stress^34^. Although it is not possible to examine these groups in depth in the current study, our findings suggest that there may be a need to investigate resilience specifically in these more vulnerable groups.

Overall, our findings have implications for the development of the ICF Core Sets for risk and resilience in developmental diversity. In particular, we identify significant heterogeneity in the factors identified by participants as influencing their risk and resilience, with only seven factors appearing in more than 50% of the interviews. This variability may present a particular challenge when developing the Core Sets, where the intention is to derive a set of ICF codes that capture risk and resilience for all neurodivergent individuals. While the overarching goal of the ICF Core Sets for resilience in neurodivergence is to provide a unified Core Set applicable to all neurodivergent individuals, and even with findings from the remaining preparatory studies being integrated, findings of the current study might suggest that a single generic set is not sufficient to capture the vast nuance within and between individuals in the neurodivergent population. This possibility was acknowledged in the initial protocol for developing these ICF Core Sets^24^. The heterogeneity observed may indicate the need for both a generic Core Set that captures common factors across neurodivergent populations, complemented by more targeted sets that capture more nuanced risk and resilience factors within specific neurodivergent groups. The possibility of generic and more targeted sets will be one question examined in the final development phase of the Core Sets, which will draw on lived and professional expertise, and the results of all four preparatory studies^24^. In addition, once developed, Core Sets must usually undergo a validation process in various contexts and populations, and be refined^23^ (e.g.,^35,36^). This step will be necessary to ensure that the sets are applicable to all neurodivergent populations, ages, and contexts (e.g., WHO regions, cultural backgrounds, country incomes). More recently, the possibility of adaptable and flexible Core Sets, building on developments in information technology, may enable individual nuances to be captured. Such approaches may be necessary in this space, where our Core Sets may serve as a starting point for more personalized versions^37^.

The emerging differences we are seeing between our three conducted preparatory studies (the review^25^, professional survey^26^, and the current study), points to both the need for Core Sets and their potential utility that these may provide in research and clinical practice. Once established, the Core Sets for risk and resilience in developmental diversity can serve as a framework for assessment, intervention, and models of service delivery, particularly when paired with other relevant approaches, such as positive psychology^21^. For example, recent work has examined how the ICF, when combined with a trauma-informed lens, can inform speech-language pathology practice^38^. By incorporating the lived experience expertise captured in the current study, the Core Set and derived assessments and intervention will better reflect the traditionally under-identified and under-emphasized factors important to neurodivergent people.

Several limitations should be noted when interpreting the findings of this study. First, although we sought to capture perspectives from individuals across different WHO regions, due to logistical limitations, the interviews were conducted only in English, Swedish, and Dutch (Flemish). Second, though we sought to comprehensively capture diverse perspectives, participants were primarily autistic or had ADHD, were, for the most part, quite highly educated, and were mostly from high-income countries. Due to these factors, our findings may not generalize to the broader neurodivergent population. In particular, individuals with other neurodivergences (such as intellectual disability) who were under-represented and individuals from low and middle-income countries, or different cultural backgrounds, may place greater or less emphasis on the importance of specific risk and resilience factors. In saying this, we still present a relatively large and diverse sample, and our findings will be combined with other preparatory studies in developing the Core Sets. Relatedly, our purpose was to generate ICF codes that captured the most pertinent risk and resilience factors for positive outcomes in neurodivergence from the perspectives of neurodivergent individuals and their loved ones. In performing this process, qualitative data is essentially quantified through linking it to the ICF system. This is a necessary step for the broader project in which this study is situated, which aims to develop ICF Core Sets for risk and resilience in neurodevelopmental diversity. Although linking this data to the ICF assists in providing evidence for key risk and resilience factors (ICF codes) that may be necessary to include in our upcoming ICF Core Set development, in turn informing assessment, intervention, and support opportunities, some nuance and personal narrative of the data is lost. Finally, as many of our participants had multiple diagnoses and given that the broader project necessitates that we investigate risk and resilience in the broader neurodivergent population, we did not perform any investigation of subgroup differences (e.g., based on diagnosis, age, WHO region). It is likely that specific nuances between different populations are not captured. Thus, future research should investigate particular nuances that may emerge within these specific groups.

## Conclusion

We explored the perspectives of neurodivergent individuals and those close to them on the factors they believe are important for risk and resilience for positive mental health, well-being, and functioning outcomes. A range of individual, activity and participation, and environmental factors influencing risk and resilience were identified, operating differently across contexts and developmental periods. Findings indicate a need to examine bio-psycho-social risk and resilience influences, and not over-emphasize the role of individual factors alone. Findings also suggest that special attention should be paid to the role of support networks, engaging in recreation and leisure, empowerment, and higher-level cognitive functions. In concert with our previous studies, identified factors may provide some preliminary foundation for assessment, intervention, and support to promote positive mental health, well-being, and functioning outcomes in neurodivergent individuals.

## Methods

### Design

This work is undertaken within the context of Risk and Resilience in Developmental Diversity and Mental Health (R2D2-MH; https://www.r2d2-mh.eu/) and is part of a larger project that follows ICF Research Branch methodology to develop ICF Core Sets for risk and resilience in developmental diversity^23^. The ICF Research Branch methodology outlines a systematic process comprising four preparatory studies intended to generate candidate ICF codes; (1) a review of existing research, (2) a survey of professionals, (3) a qualitative study, (4) and a multi-domain study. Codes generated from these preparatory studies are refined to develop the final Core Set which contain a selection of ICF codes specific to risk and resilience in neurodivergence. This study is the third in the series of preparatory works—the qualitative study. The current qualitative study comprises interviews and focus groups with neurodivergent individuals and their loved ones to identify ICF codes that capture the most pertinent risk and resilience factors to positive functioning, mental health, and well-being outcomes. The authorship team is a mixed-neurotype group, comprising both neurotypical and neurodivergent researchers. Neurodivergent researchers were involved in data interpretation and manuscript preparation. Neurodivergent individuals were also involved in the preparation of data collection materials.

### Participants

A broad and heterogenous sample was sought for this study to align with requirements for ICF Core Set development guidelines. ICF Core Sets are designed to provide a selection of ICF codes that are most relevant to describing functioning for specific populations or contexts. The data collection must be broad and comprehensive to ensure that all perspectives (e.g., across age, gender, region) are reflected in the Core Set. In our case, we thus sought a broad sample across ages, genders, WHO region, and diagnosis.

In the R2D2-MH project, neurodivergence is defined as divergent neurological functioning resulting from early differences in the structural and functional maturation of the central nervous system. Within this study, it is inclusive of the presence of a neurodevelopmental condition according to the Diagnostic and Statistical Manual of Mental Disorders Fifth Edition (DSM-5) [DSM-5;^39^] or the ICD—11th Revision [ICD-11;^40^], a genetic syndrome, or a history of pre-term birth (< 36 weeks). Loved ones were caregivers, relatives, spouses, or others close to a neurodivergent person. In defining neurodivergence, we acknowledge that there are varying perspectives and no clear consensus on its scope^41^. Here, our definition aligns with prior studies conducted as part of this series of work^24–26^ and seeks to account for the common co-occurrence of these conditions^42^. In addition, all conditions emerge in early development and can have lifelong impacts on functioning. While there are specific nuances and heterogeneity within and between these populations, they may face similar risk and resilience factors. Indeed, our previous scoping review^25^, as well as investigation in related areas such as health-related quality of life, suggests that influencing factors may be similar across neurodivergent groups^20^.

Participants from all countries were eligible to participate; however, they were required to complete the interviews in either English, Dutch (Flemish), or Swedish. Potential participants were recruited via researcher networks, advocacy groups, self-help groups, social media, and clinical services. Participants were required to self-report a formal diagnosis or a history of prematurity to be eligible for participation. Loved ones were required to confirm that the individual they were reporting on also had a formal diagnosis/history of premature birth, and participants had to be at least 7 years of age (child participants participated with a caregiver). A total of 69 individuals participated, including 48 neurodivergent individuals, eight family members or loved ones of neurodivergent individuals, and 13 individuals who represented a dual perspective, being both neurodivergent themselves as well as a caregiver or loved one of a neurodivergent person. Participants were from four WHO regions (European region, Americas, Western Pacific Region, and Africa) and represented 16 countries. Most participants were from Europe (n = 45, 65%). Loved ones were primarily parents (n = 25), but some were partners (n = 3) and siblings (n = 1). Most neurodivergent participants were autistic or had ADHD, and 41% (n = 20) had more than one diagnosis. The age of diagnosis (excluding those born prematurely) varied widely, ranging from 3 to 62 years. The average age of diagnosis was 20 years (SD = 16 years), 43% of the sample had received a diagnosis by age 11, and 66% had been diagnosed by age 20. Participants were asked if they considered themselves part of a specific cultural or ethnic group. To respect participants’ identities and provide autonomy in how they identify, participants were asked to write the groups they felt they belonged to rather than being provided with fixed categories. Fifteen neurodivergent interviewees and three loved ones responded to this question. The most common responses participants provided were “White,” “Black American, “Black British,” and “African descent.” Further demographic information is displayed in Table 5.

**Table 5:** Participant demographics.

	Neurodivergent respondents n (%)	Family/loved one respondent n (%)	Loved ones reported on by participants n (%)
n	48	21	29
Age years (range)	31.2 (9 -66)	43.1 (28–58)	14.92 (7–36)
Gender identity			
Man	20 (42%)	1 (5%)	14 (48%)
Woman	26 (54%)	20 (95%)	14 (48%)
Other	2 (4%)	0 (0%)	1 (3%)
Diagnosis*			
Autism	27 (56%)	6 (29%)	18 (62%)
ADHD	27 (56%)	10 (48%)	16 (55%)
Specific learning disability (e.g., Dyslexia)	10 (21%)	2 (10%)	5 (17%)
Born prematurely	3 (6%)	2 (10%)	2 (7%)
Communication disorder	1 (2%)	0 (0%)	3 (10%)
Genetic syndromes (e.g., Down syndrome)	0 (0%)	0 (0%)	0 (0%)
Intellectual disability/Global developmental delay	1 (2%)	0 (0%)	1 (3%)
Motor disorders (e.g., Tourette’s))	1 (2%)	0 (0%)	1 (3%)
No diagnosis	0 (0%)	5 (24%)	0 (0%)
Other	7 (15%)	5 (24%)	2 (7%)
Highest level of education			
None (currently attending preschool or elementary school)	5 (10%)	0 (0%)	–
Elementary school or equivalent	1 (2%)	0 (0%)	–
Upper secondary school or equivalent	3 (6%)	0 (0%)	–
Post-secondary education, not college/university	11 (23%)	9 (43%)	–
Studies at college/university	5 (10%)	4 (19%)	–
Degree from college/university	23 (48%)	8 (38%)	–
Socioeconomic status			
Low	9 (19%)	1 (5%)	2 (7%)
Middle	33 (69%)	18 (86%)	24 (83%)
High	6 (13%)	2 (10%)	2 (7%)
Missing	–	–	1 (3%)

*Participants could select more than one option.

### Materials

A semi-structured interview guide used for both interviews and focus groups was developed to identify the bio-psycho-social factors influencing risk and resilience in neurodivergence. The interview guide was developed based on the WHO and ICF Research Branch guidelines for ICF Core Set development^23^ and adapted for the purposes of this study. Specifically, rather than focusing on functioning more generally as described in the ICF Research Branch guidelines, questions were focused on how domains may act as risk or resilience factors. For example, a recommended question in the ICF Research Branch guidelines is: “if you think about your body and mind…what does not work the way its supposed to?” whereas our version was: “How does the way your body and mind work affect your ability to live a good life?”. Questions were designed to capture the five ICF domains: body functions, body structures, activity and participation, environmental factors, and personal factors. For example, “Thinking about what you do in your everyday life, what things affect your ability to live a good life?” (activities and participation). Each question also included examples and probing questions. The preliminary interview guide was reviewed by two co-creation groups formed as part of the broader R2D2-MH project to ensure the appropriateness of the guide. Following feedback from the groups, some modifications were made to improve the understandability of the interview questions by adding prompts and examples. The interview guide is provided in the supplement. Participants were also asked to complete a socio-demographic form that captured basic self-reported demographic information.

### Data collection

Data collection took place at two sites: one in Sweden and one in Belgium. They were performed by three members of the research team, all of whom were neurotypical (one English speaker, one Dutch speaker, and one Swedish speaker). Participants were provided with information about the study and were required to provide informed consent, or guardian consent prior to participating. They were then asked to complete an online sociodemographic form before an interview was scheduled. Data were collected primarily via interviews, with three focus group interviews conducted (1 with neurodivergent adults, 1 with relatives of neurodivergent individuals, and 1 with neurodivergent children and a caregiver). Interviews and focus groups were conducted in English (n = 36), Dutch (n = 17), and Swedish (n = 8). Interviews and focus groups were mostly conducted online to suit participants’ preferences and logistical requirements (e.g., as data were collected with participants across multiple countries). For all interviews and focus groups, efforts were made to accommodate participants’ preferences and needs. This included providing written questions in the chat when necessary, allowing breaks, conducting interviews over multiple sessions, permitting participants to turn off their cameras, and engaging in self-regulating behaviors such as stimming or engaging in different activities (e.g., crocheting). Interviews and focus groups were conducted online and lasted on average 58 min (min: 18 min, max: 1 h 47 min). The length of interviews varied due to the heterogeneity of participant needs. Other interview questions related to the conceptualization of resilience and perspectives on a good life were also asked but are not reported here.

### Data analysis

Qualitative data were transcribed verbatim and carefully read and re-read to ensure familiarity with the data. Next, an established two-phased approach for linking qualitative data to the ICF system was undertaken^43^, as commonly applied in ICF-related research [For example,^44–47^]. In phase one, we performed a qualitative content analysis approach to develop meaningful concepts that would be linked to the ICF system. First, the transcripts were segmented into units of text, which were then used to develop meaningful concepts, seeking to capture the essence or meaning of the unit. For example, the statement “Well, I would honestly say it’s probably his family and friends” was translated into two meaningful concepts: “family” and “friends.” Meaningful concepts were subsequently linked to the ICF Child and Youth (-CY) version^48^. We selected the ICF-CY because it is the most comprehensive version of the ICF, encompassing all codes in the ICF and additional codes relevant to developing individuals. In addition, as personal factors are not officially coded in the ICF but were anticipated to be relevant to risk and resilience, we also linked concepts to the supplementary personal factors classification system provided by Grotkamp et al.^49^. Linking was undertaken in accordance with the linking rules provided by the WHO and ICF research branch^43^. The most precise ICF-CY or personal factor code was applied where possible. For example, the meaningful concepts of “family” and “friends” were linked to the ICF codes e310 (immediate family) and 320 (friends), respectively. Where it was not possible to link data to an ICF-code we apply “not definable (ND)”, “not codable (NC)” or “health condition” codes to represent when there was insufficient information to apply a code, the concept was not covered the ICF(-CY) or personal factor classification system or referred to a specific diagnosis or health condition respectively. ICF coding was undertaken primarily by one researcher who was familiar with the ICF and had received ICF-linking training from the WHO ICF Research Branch. A secondary researcher also developed meaningful concepts and performed ICF-linking on a subset of the interviews to ensure the accuracy of the concept development and linking process. A third researcher with extensive experience using the ICF and ICF-linking oversaw the entire process and was available for guidance and disagreement resolution when required.

A frequency analysis was performed to summarize the findings and identify the most salient risk and resilience factors reported by the participants. Results are presented to the second level of codes, and both the absolute number of responses in which the ICF codes were identified, as well as the relative percentage, are reported. We focus on ICF codes that could be linked to the contents of at least 5% of the interviews. If responses from an individual participant could be linked to the same second-level category twice or more, they were counted only once. Although our primary aim was to capture ICF codes relevant to risk and resilience in neurodivergent populations, we also provide illustrative quotes to assist in capturing and highlighting the voices and experiences of our participants.

## Supplementary Information

Below is the link to the electronic supplementary material.

Supplementary Material 1

## Data Availability

De-identified data and ICF coding is available upon reasonable request from the corresponding author.
