# Supplementary material for: A qualitative study of risk and resilience for positive life outcomes in neurodivergence using the WHO ICF
Source: Sci Rep. 2025 Oct 9;15:35285. doi: 10.1038/s41598-025-19154-9 (PMC12511283; doi:10.1038/s41598-025-19154-9)
Supplement: Supplementary file 1 — Supplementary Material 1 [file 41598_2025_19154_MOESM1_ESM.pdf]

Resilience in Neurodivergence

ICF Semi-Structured Interview Guide

1. How does the way your body and mind work affect your ability to live a good life? (Body functions)

Examples/probing questions: Examples include your memory, your attention, how you think, your personality, or how you perceive information from your senses (sight, sound, taste, smell, etc.).

What helps you? What would help you? In what way?

What hinders you? In what way?

2. What parts of your body affect your ability to live a good life? (Body structure)

Example/probing questions: This can include things like your brain and the structure of your bones and your body.

What helps you? What would help you? In what way?

What hinders you? In what way?

3. Thinking about what you do in your everyday life, what things affect your ability to live a good life?

Examples/probing questions: This includes things like going to school or working, creating and maintaining relationships with other people, and/or taking care of yourself.

What helps you? What would help/have helped you? In what way?

What hinders you? In what way?

4. What and/or who in your environment (for example, where you live, work, or go to school) affects your ability to live a good life?

Examples/probing questions: This includes the people around you (for example, your friends, teacher, boss, etc.) and their attitudes, but also things like the physical environment, objects, and aids and/or accommodations, and economic factors.

What helps you? What would help/have helped you? In what way?

What hinders you? In what way?

5. When you think about yourself and who you are, what will help you have a good life?

Examples/probing questions: What are the things that make you unique? If you asked someone to make a description of you, what are the things they would say? This includes things like your worldview, age, gender, etc.

What helps you? What would help/have helped you? In what way?

What hinders you? In what way?
